# Unveiling the therapeutic potential of IHMT-337 in glioma treatment: targeting the EZH2-*SLC12A5* axis

**DOI:** 10.1186/s10020-024-00857-0

**Published:** 2024-06-17

**Authors:** Hongwei Zhang, Zixuan Wang, Xiaolong Qiao, Nan Peng, Jiaxing Wu, Yinan Chen, Chuandong Cheng

**Affiliations:** 1 Anhui University of Science and Technology, Huainan, 232001 Anhui China; 2 Division of Life Sciences and Medicine, Department of Neurosurgery, Centre for Leading Medicine and Advanced Technologies of IHM, The First Affiliated Hospital of USTC, University of Science and Technology of China, Hefei, 230001 Anhui China; 3 Dalian Medical University, Dalian, 116044 Liaoning China; 4 Bengbu Medical University, Bengbu, 233000 Anhui China

## Abstract

Glioma is the most common malignant tumor of the central nervous system, with EZH2 playing a crucial regulatory role. This study further explores the abnormal expression of EZH2 and its mechanisms in regulating glioma progression. Additionally, it was found that IHMT-337 can potentially be a therapeutic agent for glioma. The prognosis, expression, and localization of EZH2 were determined using bioinformatics, IHC staining, Western blot (WB) analysis, and immunofluorescence (IF) localization. The effects of EZH2 on cell function were assessed using CCK-8 assays, Transwell assays, and wound healing assays. Public databases and RT-qPCR were utilized to identify downstream targets. The mechanisms regulating these downstream targets were elucidated using MS-PCR and WB analysis. The efficacy of IHMT-337 was demonstrated through IC50 measurements, WB analysis, and RT-qPCR. The effects of IHMT-337 on glioma cells in vitro were evaluated using Transwell assays, EdU incorporation assays, and flow cytometry. The potential of IHMT-337 as a treatment for glioma was assessed using a blood–brain barrier (BBB) model and an orthotopic glioma model. Our research confirms significantly elevated EZH2 expression in gliomas, correlating with patient prognosis. EZH2 facilitates glioma proliferation, migration, and invasion alongside promoting *SLC12A5* DNA methylation. By regulating *SLC12A5* expression, EZH2 activates the WNK1-OSR1-NKCC1 pathway, enhancing its interaction with ERM to promote glioma migration. IHMT-337 targets EZH2 in vitro to inhibit WNK1 activation, thereby suppressing glioma cell migration. Additionally, it inhibits cell proliferation and arrests the cell cycle. IHMT-337 has the potential to cross the BBB and has successfully inhibited glioma progression in vivo. This study expands our understanding of the EZH2-*SLC12A5* axis in gliomas, laying a new foundation for the clinical translation of IHMT-337 and offering new insights for precision glioma therapy.

**Supplementary Information:**

The online version contains supplementary material available at 10.1186/s10020-024-00857-0.

## Introduction

Glioma is the most common malignant primary tumor of the central nervous system (CNS). Glioblastoma (GBM) represents the most severe type, with an annual incidence rate of 5.26 cases per 100,000 individuals, resulting in approximately 17,000 new diagnoses annually (Omuro and DeAngelis 2013). The 2016 World Health Organization (WHO) classification categorized gliomas from grade I to grade IV histologically (Louis et al. 2016), and in 2021, the WHO introduced distinct molecular biomarkers for various glioma types (Louis et al. 2021). This enhances clinical practice and underscores the significance of molecular markers in diagnosis and treatment.

The primary treatment for glioma typically involves a comprehensive approach combining surgery and radiochemotherapy. However, due to glioma's cellular heterogeneity and molecular abnormalities, existing treatments, including surgery and radiochemotherapy, often yield modest results and are prone to recurrence (Bent et al. 2023). Even under maximal surgical resection and radiochemotherapy, patients with glioma exhibit a median overall survival time (OS) of approximately 15–20 months, with a 2-year survival rate of merely 33.66% (Lah et al. 2020). Presently, diverse treatment modalities for glioma are emerging, but due to the presence of the blood–brain barrier (BBB), most drugs fail to achieve satisfactory efficacy (Tellingen et al. 2015). The recent emergence of targeted immunotherapy has effectively surmounted the BBB barrier, leading to significant breakthroughs in glioma treatment (Yang et al. 2022; Xun et al. 2021). However, existing molecular targets do not fully meet clinical needs. Hence, identifying additional potential molecular targets for glioma characteristics is imperative. This would form the basis for developing drugs targeting glioma with enhanced BBB penetration capabilities, thereby improving survival rates and enhancing the quality of life for glioma patients—a pressing issue needing resolution (Coy et al. 2024).

Enhancer of zeste homolog 2 (EZH2) is a histone methyltransferase that regulates target gene expression through epigenetic modifications (Duan et al. 2020). The human EZH2 gene on chromosome 7 at 7q35 comprises 20 exons and 19 introns, encoding a 746 amino acid protein (Cardoso et al. 2000). EZH2 primarily operates by forming the PRC2 functional complex with Embryonic Ectoderm Development (EED) and Suppressor of Zeste 12 Homolog (SUZ12), thereby regulating transcription. Serving as a catalytic subunit, EZH2 catalyzes H3K27 methylation, leading to trimethylation of lysine 27 on histone H3 (H3K27me3), thereby modulating downstream gene expression to maintain normal cellular function (Laugesen et al. 2019). However, when EZH2 is overexpressed, it upregulates H3K27me3 expression, silencing downstream tumor suppressor genes and inducing cellular carcinogenesis (Chang et al. 2011). Aberrant EZH2 expression or mutations play pivotal roles in tumor malignant progression, exhibiting high expression in various solid tumors such as glioma and breast cancer, thereby promoting tumor occurrence and development (Paskeh et al. 2022). Overexpression of EZH2 can suppress the expression of oncogenes like c-myc and AKT in glioma cells, thereby promoting tumor progression (Cheng and Xu 2018). Notably, recent studies suggest that the upregulation of EZH2 and myc may be induced by human cytomegalovirus (HCMV) infection (Baba et al. 2023). HCMV infection is widespread in human cancers and may drive glioma progression (Cobbs 2019; Peredo-Harvey et al. 2021).EZH2 also regulates glioma cells' metabolism and immune processes (Long et al. 2023; Xu et al. 2022). EZH2's involvement in different glioma development stages suggests its potential as a new therapeutic target for improving patient prognosis.

*SLC12A5*, a crucial member of the solute carrier family 12 subgroups, is located on human chromosome 20q13.12, a common amplification region in various malignant tumors (Doshina et al. 2017). *SLC12A5* encodes the KCC2 protein, initially identified as a transmembrane chloride and potassium transporter. This transporter aids in maintaining chloride balance inside and outside neurons, leading initial research on *SLC12A5* to focus on neurological disorders like epilepsy (Merner et al. 2015). In recent years, studies have revealed that mutations or abnormal *SLC12A5* expression contribute to the development of certain solid tumors (Kursan et al. 2017). *SLC12A5* expression promotes prostate and liver cancer progression (Yuan et al. 2023; Tong et al. 2023) while inhibiting glioma cell proliferation (Chen et al. 2023). Although few studies have reported its potential role in glioma progression, further research is warranted (Tang et al. 2020; Yang et al. 2020a).

IHMT-337, an effective EZH2-targeting inhibitor developed by our team, binds covalently to EZH2 at Cys663, promoting EZH2 degradation via the ubiquitination pathway mediated by the E3 ligase CHIP. This compound has shown promise in clinical applications by inhibiting proliferation and tumor growth of diffuse large B-cell lymphoma and triple-negative breast cancer cells in various in vitro and in vivo preclinical models (Mei et al. 2023). However, its efficacy in glioma and its ability to penetrate the BBB requires further exploration.

In conclusion, this study reaffirms the relationship between EZH2 and glioma progression, revealing EZH2's role in regulating *SLC12A5* expression by methylating its promoter region, thereby impacting glioma progression. Furthermore, it validates IHMT-337's potential to penetrate the BBB and its efficacy in treating glioma in vitro and in vivo.

## Materials and methods

### Glioma sample collection

All glioma and non-tumor brain tissues were obtained from the Department of Neurosurgery, the First Affiliated Hospital of the University of Science and Technology of China. Approval was obtained from the hospital's ethics review committee, and informed consent was obtained from all patients. Tissue samples were immediately washed several times with PBS after surgical resection until blood was roughly cleared. They were then aliquoted and stored in tubes at − 80 °C for subsequent experiments. Three senior pathologists assessed the glioma tissue used in this study to confirm the diagnosis. Non-tumor brain tissue was obtained from patients with trauma and epilepsy.

### Cell culture and transfection

The glioma cell lines U87, T98G, LN229, and U251, as well as human brain microvascular endothelial cells hCMEC/D3, were all purchased from ATCC. Human brain astrocytes (HEB), used as standard control, were obtained from our laboratory and authenticated by STR profiling. Cells were cultured in DMEM basal medium supplemented with 10% fetal bovine serum (FBS) and maintained at 37 °C in a cell culture incubator with 5% CO_2_. For transient overexpression experiments, the target gene's full-length coding sequence (CDS) was cloned into the expression vector using restriction enzymes and DNA ligase. The recombinant plasmids were then transfected into cells using transfection reagents, followed by subsequent experiments. For stable overexpression, the expression vectors were transfected into 293 T packaging cells to generate lentiviral particles, which were then used to infect target cells. Stable transfected cell lines were selected using puromycin. All plasmids were purchased from Nanjing Corues Biotech. All siRNAs used in this study were also obtained from the same company.

### Immunohistochemical (IHC) analysis

The obtained glioma and non-tumor brain tissue were fixed in formalin and sectioned into five μm thick slices. After dewaxing, endogenous peroxidase blocking, antigen retrieval, and serum blocking, the sections were incubated overnight with the corresponding primary antibodies. Subsequently, they were incubated with HRP-conjugated secondary antibodies, and DAB was used for chromogenic visualization, while hematoxylin was used to label cell nuclei. After completing all the steps, the sections were dehydrated, mounted on slides, and observed under a microscope. Five random fields were selected for photography and subsequent analysis. (The antibodies used in this step and those used in the following experimental steps can be found in Supplementary Table 1;The information regarding the specimens used in this step can be found in Supplementary Table 3).

### Western blot (WB) analysis

Following sample collection, tissues, and cells were lysed using RIPA lysis buffer (cell membrane protein extraction was conducted using a kit from Shanghai Sangon Biotech according to the manufacturer's instructions). After protein extraction, protein concentration was quantified using the BCA method (membrane protein concentration was determined using the Bradford method). Proteins were denatured by adding a loading buffer and heating at 100 °C. Subsequently, proteins were loaded onto gels for electrophoresis, and after electrophoresis, proteins were transferred onto PVDF membranes. The membranes were then blocked using 5% milk and incubated overnight at 4 °C with primary antibodies. After incubation with HRP-conjugated secondary antibodies, protein bands were visualized. All band intensities were standardized and analyzed for comparison.

### Real-time quantitative PCR (RT-qPCR) analysis

Following sample collection, total RNA was extracted from cells using the Trizol method. After determining the concentration, reverse transcription was performed to convert RNA into cDNA. Subsequently, the reaction system was prepared according to the instructions of the fluorescent quantitative PCR kit, and the samples were subjected to PCR detection according to the protocol. The reaction program was set according to the instructions. The relevant data were statistically processed and analyzed after obtaining amplification curves for the target genes. (The primer sequences for the target genes used in this study can be found in Supplementary Table 2).

### Fluorescence localization analysis

Cells in the logarithmic growth phase were evenly seeded onto confocal dishes. After adhering to the dish, cells were fixed with 4% formaldehyde, and Triton-X-100 was used to permeabilize the cell membrane. Goat serum was then used to block nonspecific sites. The primary antibodies were incubated at room temperature, followed by incubation with fluorescent secondary antibodies, and cells were stained with DAPI to label the nuclei. After completing all steps, cells were observed and photographed under a confocal microscope.

### Cell proliferation analysis

Cell proliferation was analyzed using the CCK-8 assay and EdU assay. After treatment, cells were evenly seeded into 96-well plates, and CCK-8 reagent was added at 0 h, 24 h, 48 h, and 72 h. After 1 h of incubation at 37 °C, the absorbance was analyzed using a microplate reader. The EdU assay for detecting cell proliferation was performed according to the manufacturer's instructions (Beyotime Biotech Inc., China). After staining, images were captured and analyzed under a fluorescence microscope.

### Cell migration analysis

Cell migration ability was assessed using the Transwell and scratch wound healing assays. Cells were seeded into the upper chamber of Transwell inserts after treatment for the Transwell assay and cultured in a serum-free medium. In contrast, a complete culture medium was added to the lower chamber. After 48 h of migration, cells in the upper chamber were fixed with 4% formaldehyde, stained with crystal violet, air-dried, and observed and photographed under a microscope for analysis. For the scratch wound healing assay, treated cells were evenly seeded into 6-well plates and allowed to adhere completely. A scratch was made in the cell monolayer, and cells were cultured in a serum-free medium. At 0 h, 24 h, and 48 h, cells were observed under a microscope, photographed, and analyzed.

### Cell invasion analysis

Cell invasion ability was assessed using the Transwell assay. Matrigel was evenly coated onto the upper chamber of Transwell inserts and allowed to solidify. After that, cells were seeded into the upper chamber in a serum-free medium while a complete culture medium was added to the lower chamber. After 48 h of invasion, the inserts were removed, fixed, stained with crystal violet, and observed under a microscope for photography and analysis.

### Cell apoptosis analysis

Cell apoptosis was detected using the TUNEL assay and flow cytometry. TUNEL staining was performed according to the manufacturer's instructions (Beyotime Biotech Inc., China). After staining, images were captured and analyzed under a fluorescence microscope. Following cell treatment, cells were digested, resuspended in buffer, stained with FITC Annexin V and PI, and analyzed by flow cytometry.

### Cell cycle analysis

Cell cycle analysis was performed using flow cytometry. After treatment, cells were digested and resuspended in 75% ethanol and stored at four °C overnight. Subsequently, cells were stained with propidium iodide (PI) and analyzed by flow cytometry. The data were then analyzed.

### Bioinformatics analysis

The gene expression data, gene correlation data, and survival prognosis information in this study were obtained from the TCGA database (https://www.cancer.gov/ccg/research/genome-sequencing/tcga) and the CGGA database (http://www.cgga.org.cn/). The gene expression data for normal brain tissue were sourced from the GTEx database (www.genome.gov). R language was utilized to integrate and analyze the data from these databases. Transcriptome-related analysis data were obtained from the GEO database (https://www.ncbi.nlm.nih.gov/geo/). Differential gene analysis was conducted using R language, and differential genes were annotated and subjected to enrichment analysis. Methylation-related analysis was performed using data from the TCGA database, analyzed through the GlioVis (http://gliovis.bioinfo.cnio.es/) and Methsurv (https://biit.cs.ut.ee/methsurv/) websites. Single-cell datasets (GSE138794) were obtained, and CellRanger (version 3.0.2) was used to preprocess ScRNA-seq data, assess somatic mutations, perform dimensionality reduction, calculate stemness scores, deconvolve data using linear models, reconstruct lineage through RNA velocity, and ultimately calculate relevant gene expression levels (Wang et al. 2019).

### Methylation-specific PCR analysis (MS-PCR)

MethPrimer (https://www.urogene.org/methprimer/) was used to predict CpG islands located 2000 bp upstream and 1000 bp downstream of the *SLC12A5* gene and to design primers for MS-PCR. Cells were collected and processed, and genomic DNA was extracted. After extraction, DNA purity and concentration were measured. Equal amounts of genomic DNA were treated with sodium bisulfite, followed by PCR amplification. The amplified products were then subjected to electrophoresis. After electrophoresis, the gel was exposed to a gel documentation system for imaging, and subsequent analysis was performed.

### Immunoprecipitation analysis (IP)

IP experiments were conducted using an IP kit. After sample collection, cells were lysed with lysis buffer, and proteins were collected. Protein concentration was quantified, and IP and IgG groups were set up separately. After adjusting the protein concentrations in each group, an equal amount of antibody was added to precipitate the target proteins, which were then subjected to WB analysis.

### Determination of half-maximal inhibitory concentration (IC50) of IHMT-337

The IC50 of IHMT-337 was determined using the CCK-8 assay. Cells were evenly seeded into a 96-well plate, and different concentrations of IHMT-3337 were added to the wells. After 48 h of incubation, the cells were removed, and CCK-8 reagent was added and incubated at room temperature for 1 h. Subsequently, the absorbance was measured using a microplate reader. The obtained data were then imported into GraphPad Prism 9 to calculate the IC50 value.

### Determination of the maximum absorption peak of IHMT-337

The maximum absorption peak of IHMT-337 was determined using spectrophotometry. IHMT-337 was uniformly dissolved in DMEM to prepare solutions with concentrations of 0 μM (control), 1 μM, 5 μM, and 10 μM, each with a volume of 10 ml. After calibrating the NanoDrop 2000, the prepared solutions were dropped into the instrument for reading, and after multiple experiments, the position of the maximum absorption peak was determined.

### Construction of an in vitro BBB model and analysis of IHMT-337 permeability

hCMEC/D3 cells were cultured in a DMEM medium containing 15% FBS until reaching 70% confluence, followed by passaging. The hCMEC/D3 cells were seeded onto the upper chamber of Transwell inserts and cultured in DMEM medium containing 15% FBS for 3–5 days until completely confluent. After confirming cell confluency, the barrier integrity was assessed. To measure barrier integrity in the apical-to-basolateral direction, fluorescently labeled 70 kDa or 4 kDa dextran (final concentration of 1 μM) was added to the upper chamber (total volume 100 μl). At specified time points (30 min—180 min), samples (50 μl) were collected from the basolateral chamber (total volume 600 μl) and replaced with an equal volume of medium to compensate for the volume reduction caused by sampling. To measure barrier integrity in the basolateral-to-apical direction, fluorescently labeled dextran (70 kDa or 4 kDa, final concentration of 1 μM) was added to the basolateral chamber, and samples (10 μl) were collected from the apical chamber, followed by replacement with medium to compensate for the volume reduction due to sampling (Biemans et al. 2017). After confirming barrier integrity, different concentrations of IHMT-337 were added to the upper chamber. At specified time points, absorbance was measured at the maximum absorption peak, and the amount of IHMT-337 passing through was determined based on absorbance measurements. Data were recorded, and the permeability efficiency of IHMT-337 was calculated.

### Construction of orthotopic glioma model and in vivo imaging

A U87 cell line overexpressing the luciferase gene and the target gene of interest was constructed. Nude mice were anesthetized, and stereotactic surgery was performed using a brain stereotactic apparatus to inoculate the cells into the right caudate nucleus region of the nude mouse brain. Postoperatively, the wound was disinfected to ensure the survival of nude mice. One week later, the mice were anesthetized and placed in an animal in vivo imaging system to confirm tumor formation. The nude mice overexpressing EZH2 were divided into two groups: one group received an intraperitoneal injection of IHMT-337 at a dose of 100 mg/kg (Q4D), and the other group received an equal volume of solvent as control treatment. The control group also received solvent treatment. After a period of treatment, the second in vivo imaging was performed. During this period, the survival period of nude mice was recorded. Tumor-bearing nude mice were intraperitoneally injected with 100 μl of D-luciferin potassium salt (15 mg/ml), allowed to move for 5 min freely, then anesthetized with isoflurane, and imaging was performed using an in vivo imaging system.

### Statistical analysis

All experiments were performed in triplicate, and all data are presented as mean ± standard deviation (SD). Statistical analysis was conducted using GraphPad Prism 9. Statistical significance was calculated using t-tests or one-way analysis of variance (ANOVA). A *p*-value of less than 0.05 (*) was considered statistically significant.

## Results

### EZH2 is significantly upregulated in gliomas and correlates prominently with patient prognosis

**Fig. 1:**
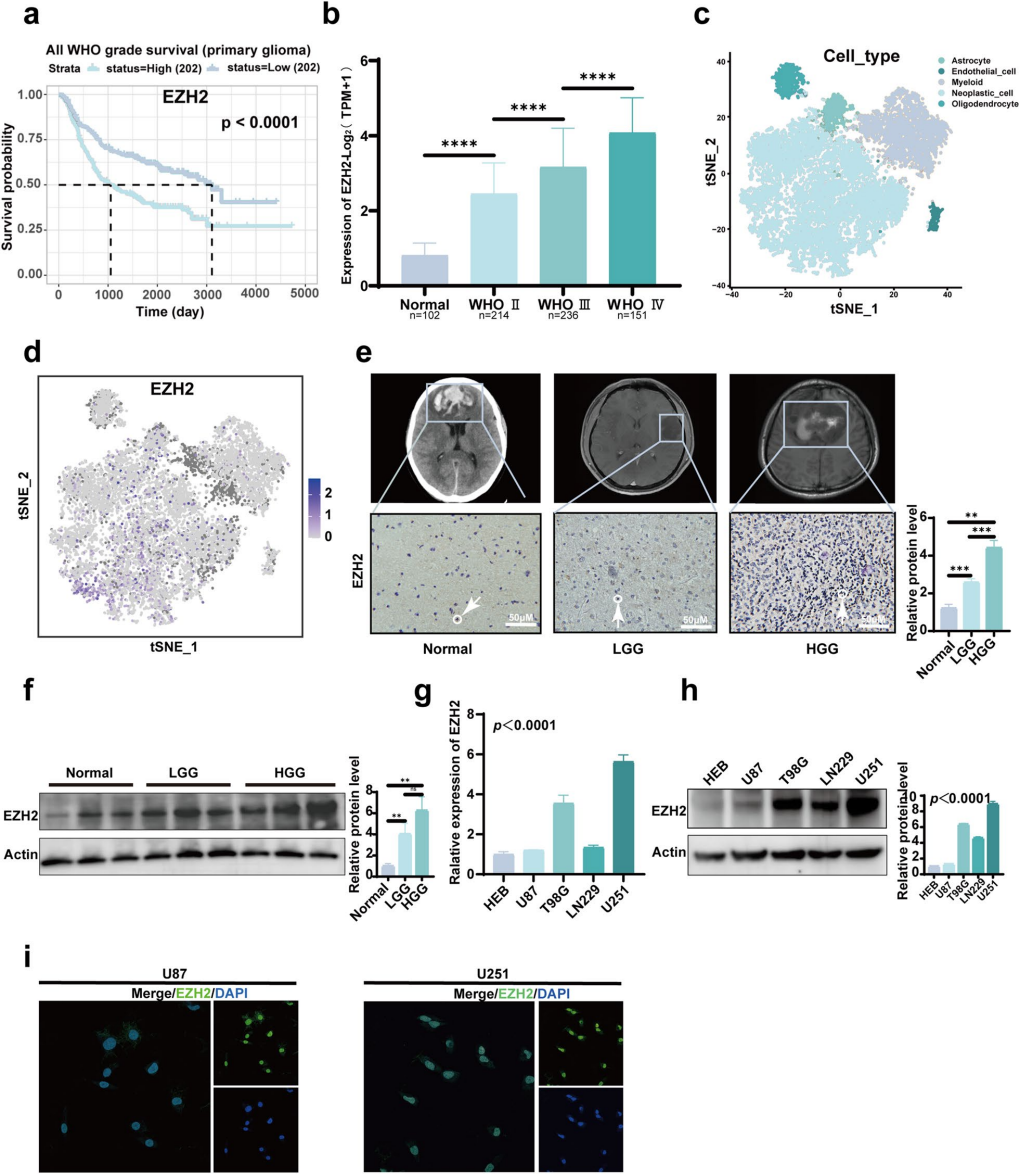
EZH2's prognosis, expression, and cellular localization. **a** According to data from the CGGA database, patients with high EZH2 expression have a poorer prognosis. **b** Integrated data from TCGA and GTEx show high expression of EZH2 in glioma tissues. **c** A public single-cell dataset results indicate differential expression of EZH2 across various cell subtypes. **d** Single-cell analysis reveals predominant expression of EZH2 in tumor cell subpopulations. **e** IHC demonstrates higher expression of EZH2 in glioma tissues compared to normal brain tissue. The images above depict corresponding radiological images of patients. **f** WB results indicate elevated expression of EZH2 in glioma tissues. **g** RT-qPCR results show higher mRNA expression of EZH2 in glioma cells compared to astrocytes. **h** WB results demonstrate higher protein expression of EZH2 in cells compared to normal astrocytes. **i** Fluorescence localization results show predominant nuclear localization of EZH2. (**p < 0.01, ***p < 0.001, ****p < 0.0001)

We validated the relationship between EZH2 expression and prognosis in both TCGA and CGGA databases, revealing that patients with high EZH2 expression (using the median expression level as the cut-off value) exhibit significantly worse prognosis compared to those with low EZH2 expression in both primary and recurrent gliomas (Fig. 1a and Fig. S1a, 1b). Integrating EZH2 expression data from TCGA and GTEx, we observed significantly elevated EZH2 expression in tumor tissues compared to control tissues, with expression levels increasing with WHO grades (Fig. 1b). Furthermore, utilizing publicly available single-cell datasets, we demonstrated that EZH2 is predominantly expressed in tumor cell populations (Fig. 1c, d). IHC staining and WB analysis confirmed the significant upregulation of EZH2 in gliomas (Fig. 1e, f). After validating EZH2 expression at the tissue level, we examined EZH2 mRNA and protein expression at the cellular level, revealing higher levels of EZH2 mRNA and protein in glioma cell lines compared to normal astrocytes, with the lowest expression in U87 and the highest expression in U251 cells, which were selected for subsequent experiments (Fig. 1g, h). Finally, we investigated the cellular localization of EZH2. We found that it is predominantly expressed in the cell nucleus (Fig. 1i). These cellular localization findings suggest a potentially crucial role for EZH2 within the nucleus.

### EZH2 promotes glioma cell proliferation, invasion, and migration

**Fig. 2:**
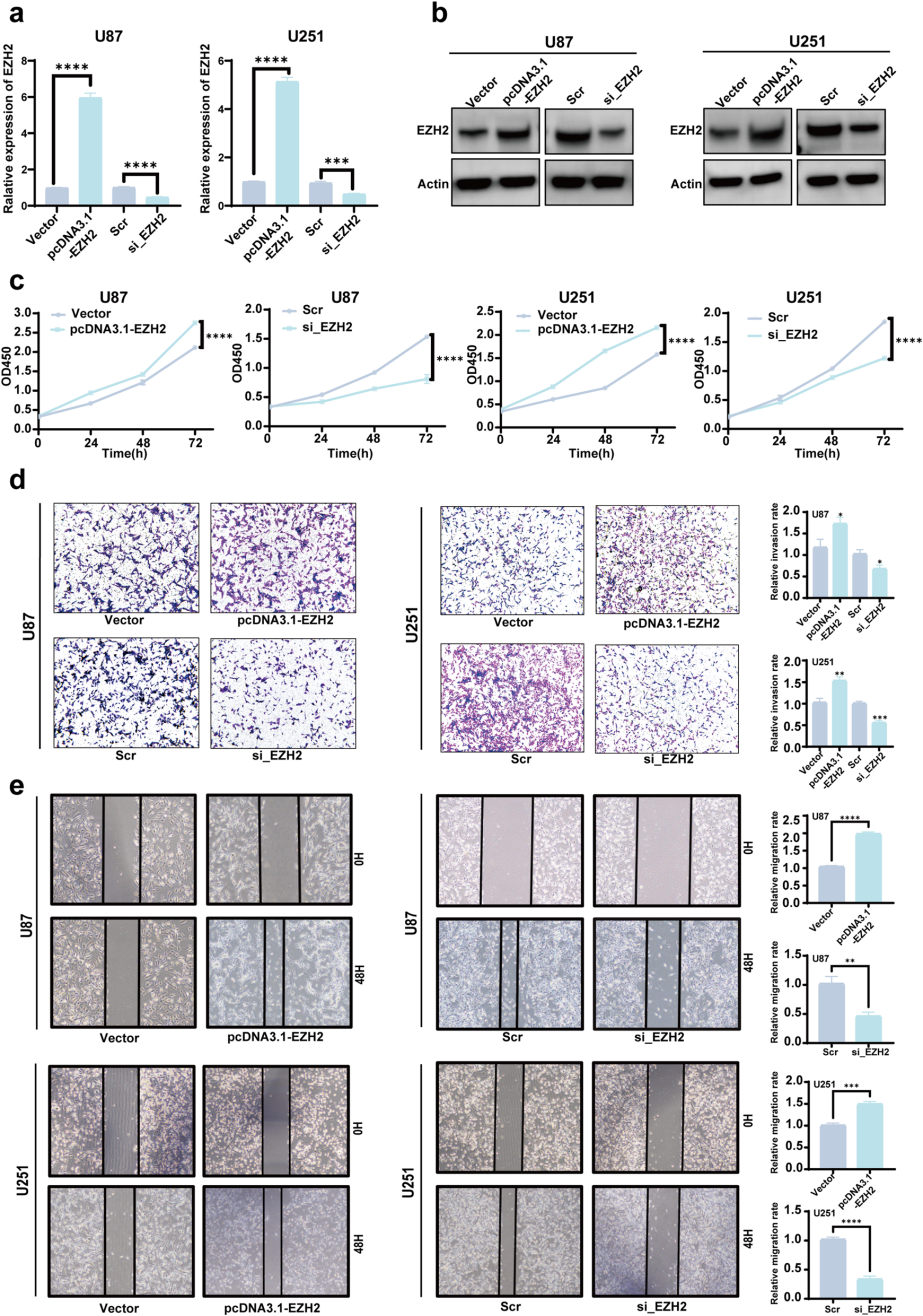
EZH2 promotes the proliferation, migration, and invasion of gliomas. **a** RT-qPCR detected mRNA expression level of EZH2 in U87 and U251 cells after overexpression or knockdown. **b** WB detected protein expression level of EZH2 in U87 and U251 cells after overexpression or knockdown. **c** CCK-8 assay detected the proliferation of U87 and U251 cells after overexpression or knockdown of EZH2. **d** Transwell assay was used to assess the invasion ability of U87 and U251 cells after overexpression or knockdown of EZH2. **e** Scratch wound healing assay was used to evaluate the migration ability of U87 and U251 cells after overexpression or knockdown of EZH2. (Vector: Normal expression of EZH2; pcDNA3.1-EZH2: High expression of EZH2; Scr: siRNA control sequence; si_EZH2: Low expression of EZH2;*p < 0.05, **p < 0.01, ***p < 0.001, ****p < 0.0001)

Based on the aforementioned experimental findings indicating high expression of EZH2 in gliomas, we further investigated the impact of EZH2 alterations on cellular functions in vitro. We transfected U87 and U251 cells with an EZH2 overexpression plasmid (pcDNA3.1-EZH2) and its control plasmid (Vector). To knock down the expression of EZH2, we transfected cells with EZH2 interfering siRNA (si_EZH2) and its control sequence (Scr). Changes at the mRNA and protein levels confirmed significant alterations in EZH2 expression (Fig. 2a, b). Upon successfully establishing EZH2 overexpression and interference, we examined the effects of EZH2 on cell proliferation. The results revealed a significant enhancement of cell proliferation upon EZH2 overexpression, whereas interference with EZH2 led to the opposite outcome (Fig. 2c). Similarly, we investigated the influence of EZH2 on cell invasion capability. It was observed that EZH2 overexpression markedly enhanced cell invasion. At the same time, interference with EZH2 resulted in a significant reduction in invasion ability (Fig. 2d). Lastly, we assessed the impact of EZH2 on cell migration ability. The results demonstrated that EZH2 overexpression augmented cell migration capability, whereas interference with EZH2 expression yielded the opposite effect (Fig. 2e).

### Differential and enrichment analysis of EZH2 downstream genes

**Fig. 3:**
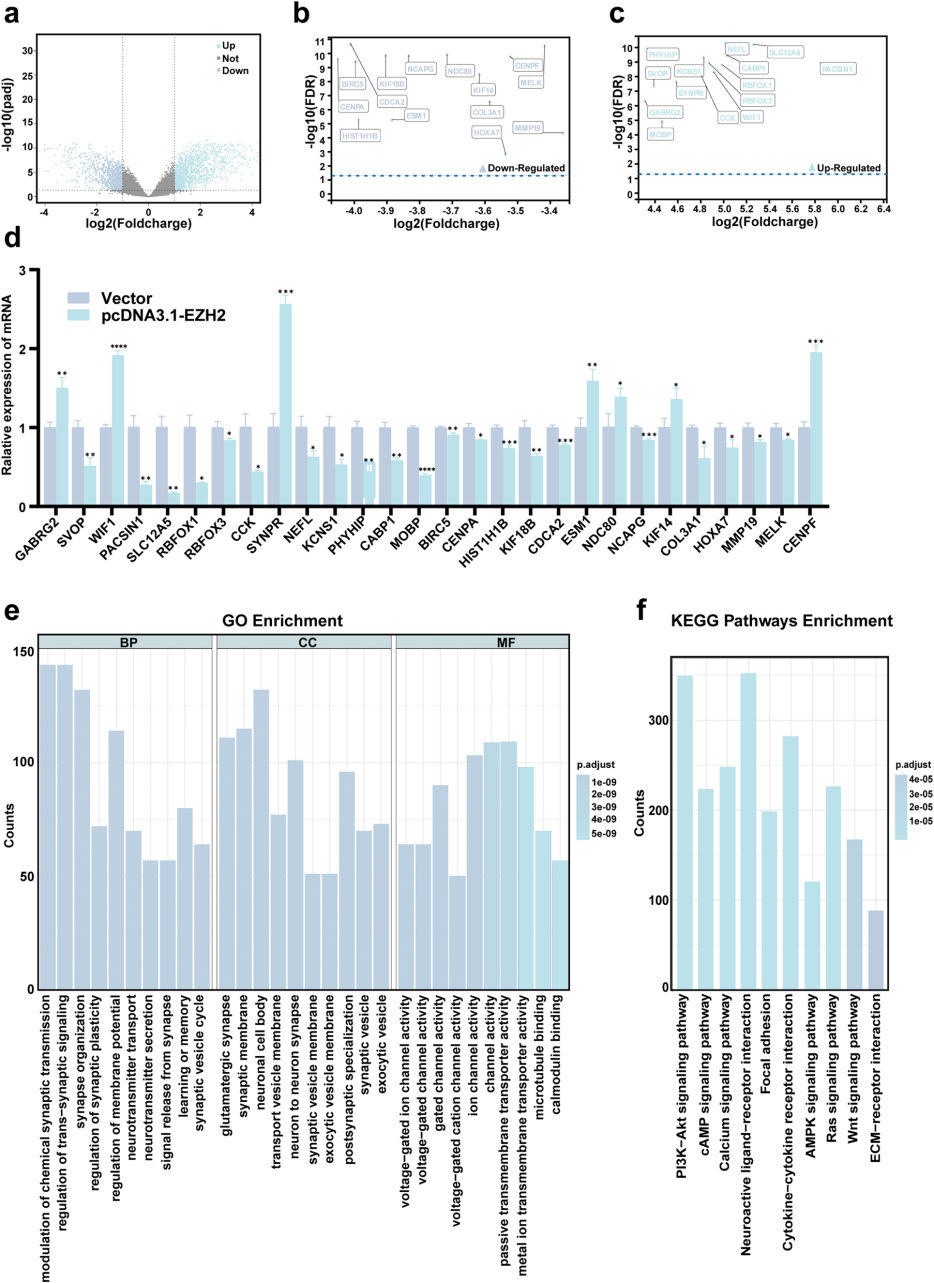
Differential and enrichment analysis of downstream genes of EZH2. **a** Volcano plot showed differential downstream genes after EZH2 alteration. **b** Most significantly upregulated genes among all differential genes. **c** Most significantly downregulated genes among all differential genes. **d** After overexpression of EZH2 in U87 cells, RT-qPCR was used to detect changes in the expression levels of the above genes. **e** GO analysis of differential genes. **f** KEGG enrichment analysis of downregulated differential genes. (Vector: Normal expression of EZH2; pcDNA3.1-EZH2: High expression of EZH2;*p < 0.05, **p < 0.01, ***p < 0.001, ****p < 0.0001)

To delve deeper into the mechanistic insights of EZH2's impact on cellular functions in vitro, we obtained a dataset (GSE221440) from the GEO database and grouped it based on EZH2 expression levels. Differential expression genes (DEGs) were selected with adjusted *p*-value < 0.05 and |logFC|> 1 considered as significantly differentially expressed genes. A volcano plot illustrating the DEGs was generated (Fig. 3a), with downregulated considerably (Fig. 3b) and upregulated (Fig. 3c) DEGs labeled. Validation of these DEGs was conducted in cell lines, revealing significant alterations in the expression of *SLC12A5* and *SYNPR* following EZH2 overexpression, which were subsequently chosen for further analysis (Fig. 3d). GO analysis was performed on all DEGs, including biological processes (BP), cellular components (CC), and molecular functions (MF). BP analysis indicated EZH2 alterations triggered regulation of chemical synaptic transmission and modulation of GABAergic synapse signaling, among other changes. CC analysis revealed alterations in neuronal cell bodies, synaptic membranes, and glutamatergic synapses. MF analysis highlighted abnormal changes in channel activity, passive transmembrane transporter activity, transporter activity, ion channel activity, and calcium ion binding (Fig. 3e). KEGG pathway enrichment analysis indicated that DEGs were mainly enriched in pathways related to PI3K-AKT, cAMP, and others (Fig. 3f). Combining the results of enrichment analysis, we found that the SLC family genes extensively participated in the above-mentioned biological processes. Considering EZH2's function, we speculate that EZH2 may directly silence the expression of *SLC12A5*. Finally, we analyzed the expression and prognosis of *SLC12A5* in TCGA and CGGA datasets, revealing low expression of *SLC12A5* in gliomas, particularly in GBM (Fig. S1c). Moreover, the prognosis of the high-expression group of *SLC12A5* was significantly better than that of the low-expression group, indicating a significant correlation between *SLC12A5* expression and prognosis in gliomas (Fig. S1d-g).

### EZH2 suppresses *SLC12A5* expression through DNA methylation

**Fig. 4:**
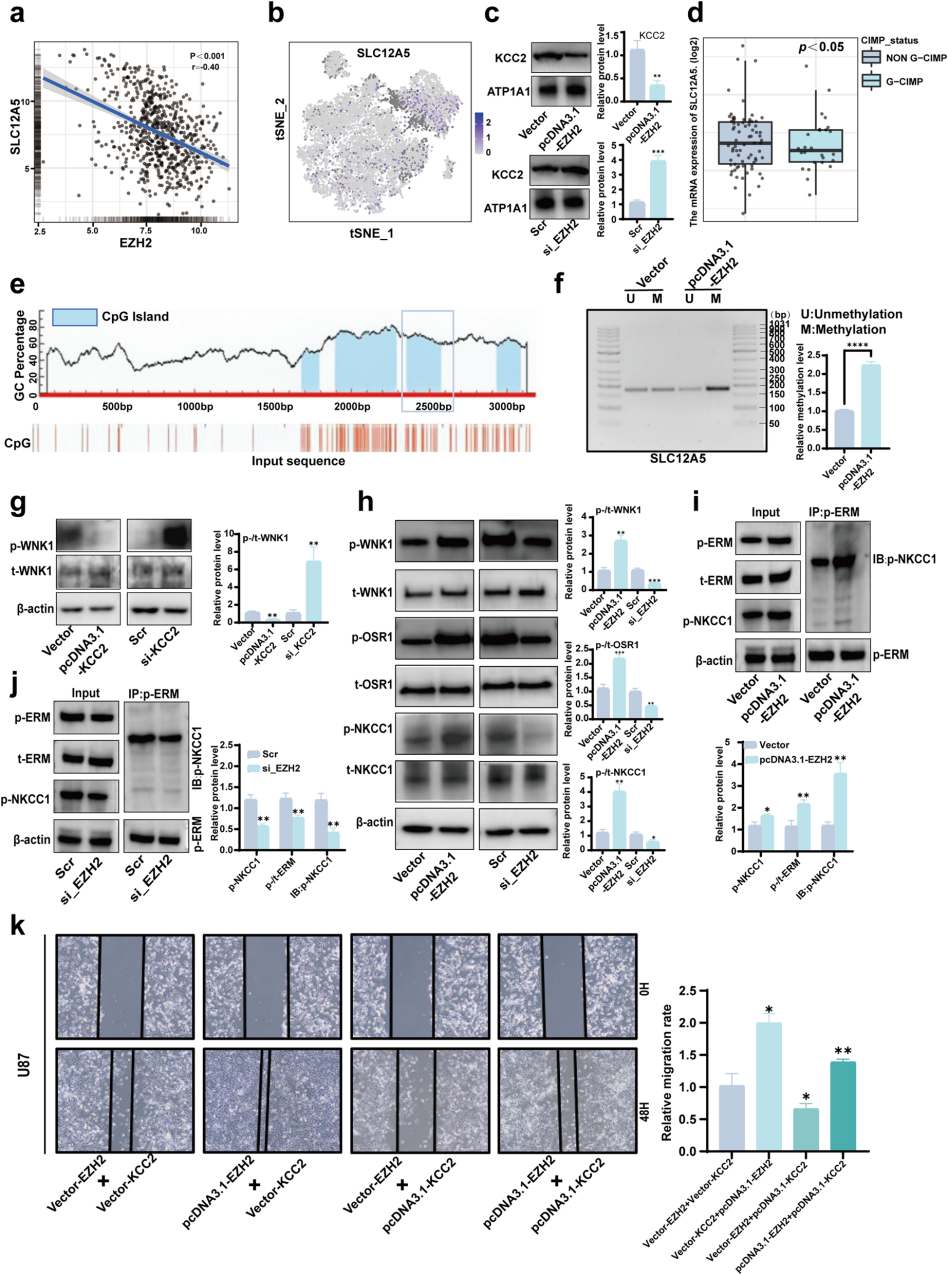
EZH2 regulates the expression of *SLC12A5* through DNA methylation and activates the WNK1-OSR1-NKCC1 cascade to control glioma migration. **a** Correlation analysis of *EZH2* and *SLC12A5* expression using integrated TCGA data. **b** The expression profile of *SLC12A5* was analyzed from single-cell data. **c** WB detected the expression of KCC2 after overexpression or knockdown of EZH2. **d** The influence of methylation status on the expression of *SLC12A5 *was analyzed using the GlioVis database. **e** Prediction of CpG islands within the first 2000 bp of *SLC12A5*'s coding sequence and its own 1000 bp. Boxes indicate regions for designing methylation primers. **f** The increased methylation level of *SLC12A5* DNA region after EZH2 overexpression in U87 cells was detected by MS-PCR. **g** Increased WNK1 activity after overexpression and knockdown of KCC2 in U87 cells; decreased WNK1 activity after knockdown of KCC2. **h** Activation of the WNK-OSR1-NKCC1 pathway after overexpression and knockdown of EZH2 in U87 cells. **i** Increased ERM protein activity after EZH2 overexpression in U87 cells; increased p-NKCC1 under the same p-ERM level. **j** Decreased ERM protein activity after knockdown of EZH2 in U87 cells; decreased p-NKCC1 under the same p-ERM level. **g** and **h** were detected by WB. **i** and **j** were detected by IP. **k** The migration ability of U87 cells overexpressing EZH2, KCC2, or co-overexpressing EZH2 and KCC2 was assessed using scratch wound healing assay. (Vector: Normal expression of EZH2 and KCC2; pcDNA3.1-EZH2: High expression of EZH2; pcDNA3.1-KCC2: High expression of KCC2; Scr: siRNA control sequence; si_EZH2: Low expression of EZH2; si_KCC2: Low expression of KCC2*;*p < 0.05, **p < 0.01, ***p < 0.001, ****p < 0.0001)

Analysis of integrated glioma data from the TCGA database revealed a negative correlation between EZH2 and *SLC12A5* expression (Fig. 4a). Results from single-cell data analysis showed that compared to tumor cell populations, *SLC12A5* expression was more abundant in non-tumor cell populations (Fig. 4b). This phenomenon was further validated at the protein level, where overexpression of EZH2 in U87 cells led to a decrease in KCC2 protein levels. In contrast, interference with EZH2 expression resulted in an elevation of KCC2 expression (Fig. 4c). Utilizing data provided by GlioVis, we grouped samples based on methylation status. We found that *SLC12A5* expression was lower in the methylated group compared to the unmethylated group (Fig. 4d), suggesting that *SLC12A5* expression may be regulated by methylation. Using MethPrimer, we predicted CpG islands in the upstream 2000 bp region of the *SLC12A5* as well as its 1000 bp region (Fig. 4e). Combining this with data analysis from Methsurv, we identified areas of high methylation in the *SLC12A5* (Fig. S2a), and designed methylation-specific primers for MS-PCR detection. The results revealed a significant increase in methylation levels in the promoter region of *SLC12A5* following EZH2 overexpression (Fig. 4f).

### EZH2 regulates KCC2 expression to influence the activity of the WNK1-OSR1-NKCC1 pathway and glioma migration

KCC2, as a crucial protein involved in intracellular Cl- regulation, has been previously reported to be influenced by Cl^−^ levels, which in turn modulate the activity of the WNK1-OSR1-NKCC1 pathway. We transfected cells with a KCC2 overexpression plasmid (pcDNA3.1-KCC2) and its control plasmid (Vector), as well as interfering siRNA targeting KCC2 (si_KCC2) and its control sequence (Scr). The results revealed a significant alteration in the activity of WNK1. (Fig. 4g and Fig. S2b). Subsequently, we examined the impact of EZH2 on the WNK1-OSR1-NKCC1 cascade and found that overexpression of EZH2 significantly activated this pathway. In contrast, interference with EZH2 suppressed its activity (Fig. 4h). Activation of NKCC1 in the path above can interact with ERM (Ezrin, Radixin, and Moesin) proteins. Therefore, we assessed the changes in ERM activity under different EZH2 expression states and performed an IP pull-down of p-NKCC1. The results showed an increase in ERM activity and interaction with p-NKCC1 following EZH2 overexpression (Fig. 4i), with the opposite effect observed upon EZH2 interference (Fig. 4j). ERM acts as a bridge between actin and p-NKCC1, and such changes may affect cell migration ability. Following these findings, we conducted rescue experiments to further confirm the impact of EZH2 and KCC2 alterations on glioma migration. The results revealed that sole overexpression of EZH2 promoted glioma migration, while sole overexpression of KCC2 inhibited glioma migration. However, simultaneous overexpression of EZH2 and KCC2 rescued the promoting effect of EZH2 on glioma migration (Fig. 4k and Fig. S2c).

### IHMT-337 inhibits glioma cell migration and proliferation and arrests the cell cycle in vitro

**Fig. 5:**
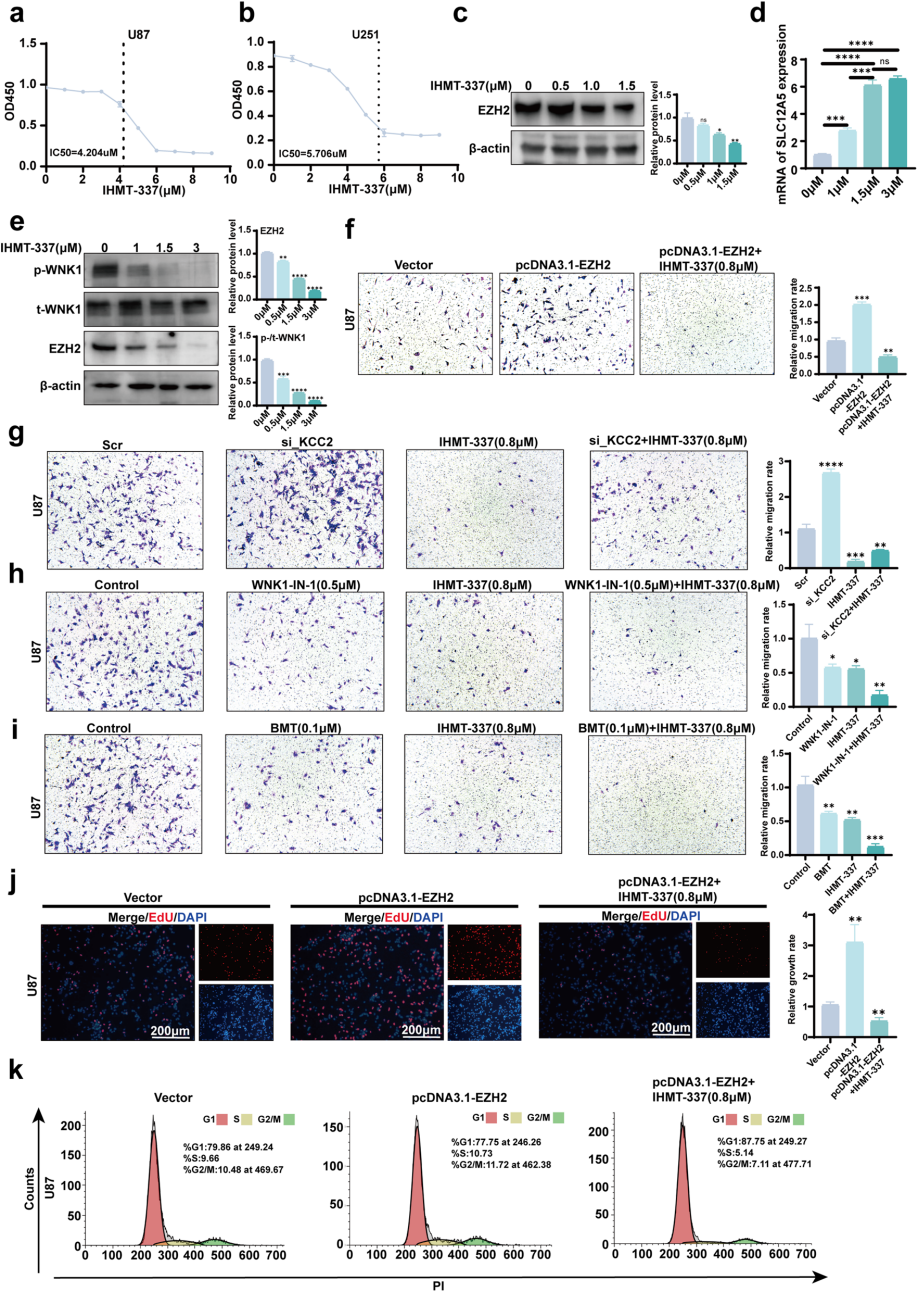
IHMT-337 targets EZH2 to inhibit WNK1 activation, thereby suppressing glioma migration. Additionally, it inhibits cell proliferation and arrests the cell cycle. **a** IC50 of IHMT-337 in U87 cells. **b** IC50 of IHMT-337 in U251 cells. **c** Degradation of EZH2 at different concentrations of IHMT-337 was detected by WB. **d** Increase in *SLC12A5* mRNA expression at different concentrations of IHMT-337 was detected by RT-qPCR. **e** Inhibition of WNK1 activity by different concentrations of IHMT-337 was detected by WB. **f** In U87 cells, IHMT-337 inhibits cell migration promoted by EZH2 overexpression. **g** In U87 cells, IHMT-337 inhibits cell migration induced by decreased KCC2 protein levels. **h** IHMT-337 synergizes with WNK1-IN-1 to inhibit glioma migration in U87 cells. **i** IHMT-337 synergizes with BMT to inhibit glioma migration in U87 cells. **f**–**i** were detected by Transwell. **j** EdU assay was used to assess the effect of IHMT-337 on cell proliferation in U87 cells. **k** Flow cytometry was used to evaluate the impact of IHMT-337 on the cell cycle in U87 cells. (Vector: Normal expression of EZH2; pcDNA3.1-EZH2: High expression of EZH2; *p < 0.05, **p < 0.01, ***p < 0.001, ****p < 0.0001)

Building upon the findings above, we aimed to inhibit the activation of the WNK1-OSR1-NKCC1 cascade and, consequently, glioma migration at its source using an EZH2 inhibitor. Initially, we determined the half-maximal inhibitory concentration (IC50) of IHMT-337 in glioma cells, revealing an IC50 of 4.204 μM in U87 cells and 5.706 μM in U251 cells (Fig. 5a,5b). Subsequently, we investigated the ability of different concentrations of IHMT-337 to degrade EZH2 in glioma cells. The results showed reduced EZH2 protein levels with increasing IHMT-337 concentrations (Fig. 5c and Fig S2d). In contrast, *SLC12A5* mRNA levels increased (Fig. 5d). Furthermore, as EZH2 protein levels decreased, WNK1 activity was gradually inhibited (Fig. 5e). We then evaluated the effect of IHMT-337 on glioma cell migration, revealing a significant inhibition of cell migration promoted by EZH2 overexpression (Fig. 5f), as well as the suppression of cell migration induced by KCC2 downregulation (Fig. 5g). Additionally, we found that IHMT-337 synergistically inhibited cell migration when combined with WNK1 inhibitor WNK1-IN-1 and NKCC1 inhibitor bumetanide (BMT) (Fig. 5h, I). We further extended the investigation into the in vitro effects of IHMT-337. The results demonstrated that IHMT-337 significantly inhibited glioma cell proliferation (Fig. 5j and Fig.S3c, S3d) and arrested the cell cycle progression (Fig. 5k and Fig.S3e). However, it did not significantly impact cell apoptosis (Fig.S3a, b).

### IHMT-337 exhibits BBB penetration in vitro and inhibits glioma growth in vivo, prolonging survival in nude mice

**Fig. 6:**
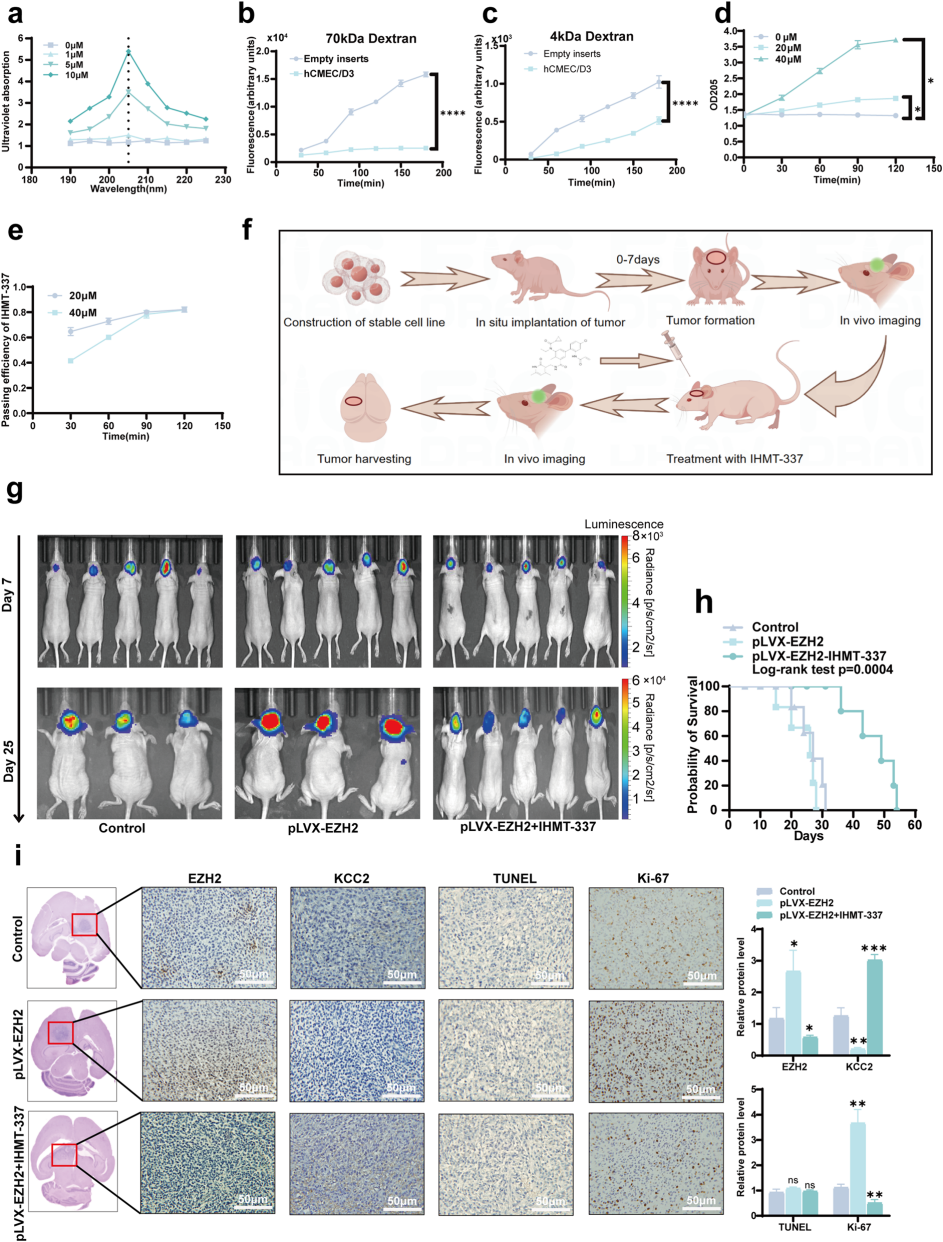
IHMT-337 demonstrates penetration through the BBB in vitro and inhibits glioma progression in vivo, prolonging the survival of nude mice. **a** Maximum UV absorption peak of IHMT-337. **b** Permeability of 70 kDa dextran across the BBB model. **c** Permeability of 4 kDa dextran across the BBB model. **d** Permeability of IHMT-337 across the BBB model. **e** The efficiency of IHMT-337 penetration through the BBB in vitro. **f** The overall process of in vivo experiments with IHMT-337 in nude mice. **g** Live imaging of animal models in each group. **h** Survival period of nude mice in each group. **i** H&E detected the tumor location of  the  nude mice, IHC staining was used to detect the expression of EZH2, KCC2 and Ki-67, and TUNEL analysed apoptosis-related protein expression. (pLVX-EZH2: high expression of EZH2; *p < 0.05, **p < 0.01, ***p < 0.001, ****p < 0.0001)

Initially, we determined the maximum UV absorption peak of IHMT-337, revealing a peak at 205 nm (Fig. 6a). Next, we established an in vitro BBB model and assessed its integrity. Comparison between blank pores and barrier pores showed robust formation of the barrier for the 70 kDa dextran group (Fig. 6b). In contrast, significantly lower barrier formation was observed for the four kDa group (Fig. 6c). Subsequently, we verified the ability of IHMT-337 to penetrate the BBB in vitro, detecting the presence of IHMT-337 in the lower chamber of the BBB model (Fig. 6d), with an approximate transmittance efficiency of 82% after 90 min (Fig. 6e). Based on these results, we established an in situ glioma model in nude mice and treated gliomas with IHMT-337 (Fig. 6f). Initially, we engineered stable cell lines carrying luciferase genes and overexpressing EZH2, along with a control group, which were implanted intracranially into mice. Tumors were observed to form significantly after seven days. Subsequently, the control group, EZH2 overexpression group, and treatment group were administered physiological saline, physiological saline, and IHMT-337, respectively. Results from the second imaging session on day 25 showed significantly larger tumors in the EZH2 overexpression group compared to the control and treatment groups. In comparison, tumors in the treatment group were notably smaller than those in the control and EZH2 overexpression groups (Fig. 6g). The survival period of the mice was recorded during this time, revealing a significantly more extended survival period in the treatment group compared to the control and EZH2 overexpression groups (Fig. 6h). Finally, H&E staining of mouse brain tissue confirmed tumor location, followed by IHC staining, which demonstrated significantly lower EZH2 expression and significantly higher KCC2 expression in the treatment group compared to the other two groups. The expression of Ki-67 in the treatment group was significantly lower than in the other two groups. However, there was almost no difference in TUNEL staining among the three groups. These findings indicate that IHMT-337 can inhibit glioma proliferation in vivo but does not significantly affect glioma cells' apoptosis (Fig. 6i).

## Discussion

EZH2 is a protein significantly upregulated in gliomas and can promote malignant progression through various mechanisms (Kim et al. 2013; Yang et al. 2020b; Xu et al. 2019). Recent reports indicate that the high expression of EZH2 in gliomas is attributed to HCMV infection, and successful induction of GBM was achieved in vitro models by transplanting HCMV-infected astrocytes (Guyon 2024). Gliomas also demonstrate sensitivity to EZH2 inhibitors, TMZ, and ganciclovir combination therapy, further emphasizing the importance of EZH2 in gliomas (Baba et al. 2023). We validated the abnormally high expression of EZH2 in gliomas, which promotes proliferation, migration, and invasion of glioma cells. Utilizing publicly available transcriptomic datasets, we identified downstream genes of EZH2 and the biological processes in which it participates. Following EZH2 overexpression, significant changes were observed in the mRNA levels of *SYNPR* and *SLC12A5*. One of the crucial mechanisms by which EZH2 promotes tumor progression is as a catalytic subunit of the PRC2 complex, which silences tumor suppressor gene transcription by forming H3K27me3 (Margueron and Reinberg 2011). Therefore, we chose *SLC12A5* for further study.DNA methylation-mediated gene silencing is an essential mechanism of gene expression regulation in eukaryotic cells (Dong et al. 2018). Previous reports have demonstrated that EZH2 can regulate DNA methylation in lupus patients and control T cell adhesion by connecting with adhesion molecule A (Tsou et al. 2018). DNA methylation in mammalian cells primarily occurs on cytosines (C) in CpG dinucleotides and is relatively stable during mitosis, crucial for maintaining cell lineage characteristics (Ming et al. 2020). Therefore, we hypothesized that EZH2 may suppress *SLC12A5* expression by inducing DNA methylation. To validate our hypothesis, we utilized database data to confirm the presence of numerous methylated sites in the promoter region of *SLC12A5*. Furthermore, the prediction of CpG islands upstream of the *SLC12A5* gene and combined analysis with Methsurv led us to select primers targeting a CpG island located 1500 bp upstream of the transcription start site for verification, further confirming our hypothesis.

We predicted the relationship between *SLC12A5* and glioma progression using data from multiple databases, and the results revealed a significant correlation between the expression of *SLC12A5* and prognosis. According to previous literature, KCC2 is regulated by the WNK1 protein (Gao et al. 2019). The WNK family of protein kinases plays a crucial role in regulating Na^+^, K^+^, and Cl^−^ ions by modulating downstream kinases SPAK/OSR1 and their target NKCC1. The mammalian genome encodes four homologs of WNK, namely WNK1, WNK2, WNK3, and WNK4. WNK1 (Veríssimo and Jordan 2001), a widely expressed serine-threonine kinase, is known to modulate ion homeostasis, with much research focusing on its relationship with hypertension and other human diseases (Meor Azlan et al. 2021; Shekarabi et al. 2017). Reports suggest that ion concentrations can regulate WNK activity. Intracellular Cl − directly interacts with the active site of WNK, inhibiting WNK autophosphorylation and activation. Under low intracellular [Cl^−^]_i_ conditions, inhibition of WNK1 by Cl^−^ is relieved, leading to WNK1 activation (Piala, et al. 2014). The crucial protein regulating intracellular Cl^−^ concentration is encoded by *SLC12A5*, which encodes the KCC2 protein responsible for extruding Cl^−^ from the cell, while *SLC12A2* encodes the NKCC1 protein accountable for transporting Cl^−^ into the cell (Liu et al. 2019). NKCC1 is widely expressed in both brain and non-brain cells (Kurki et al. 2023), while KCC2 is primarily expressed in central nervous system structures (Markkanen et al. 2014; Vu et al. 2000; Hübner et al. 2001). Under physiological conditions, intracellular chloride ion concentration ([Cl^−^]_i_) is much lower than extracellular chloride ion concentration ([Cl^−^]_o_) (Ben-Ari 2002), primarily due to the relatively stable levels of these two proteins. However, our study found that EZH2 significantly inhibits the expression of *SLC12A5*, affecting the balance of these two proteins. Consequently, we assessed the expression of KCC2 protein and found it reduced. Furthermore, we observed that WNK1 activity is significantly inhibited when *KCC2* is overexpressed, while the opposite result occurs when *KCC2* expression is suppressed. Previous studies have shown that T cell migration is associated with WNK1 activation, which opens "leading edge" channels, increasing water and ions and promoting cell migration (Schwab et al. 2012; Xu et al. 2002). WNK1 activates the WNK1-OSR1-NKCC1 cascade to regulate glioma cell migration, where certain stimuli (such as cell contraction and hyperosmolarity) can induce phosphorylation of the WNK1-OSR1-NKCC pathway in GBM. Phosphorylated NKCC can interact with the ezrin-radixin-moesin (ERM) protein family, leading to cytoskeletal rearrangement and promoting cell migration (Zhu et al. 2014). Therefore, we believe that EZH2 can regulate the expression of SLC12A5 to activate the WNK1-OSR1-NKCC1 cascade, which controls the migration of glioma cells. We examined the activation status of this pathway after EZH2 overexpression and found that the path was significantly activated. Conversely, when EZH2 was disrupted, the pathway was inhibited. Further experiments revealed that overexpression of EZH2 promoted cell migration, while overexpression of SLC12A5 inhibited cell migration. Moreover, when both EZH2 and SLC12A5 were overexpressed, the degree of cell migration was reduced compared to overexpression of EZH2 alone. Therefore, we believe that EZH2 can inhibit glioma migration by regulating the expression of SLC12A5, and the mechanism underlying this phenomenon is likely due to the activation of the WNK1-OSR1-NKCC1 cascade.

The balance between excitation and inhibition (E/I balance) in the brain is crucial for maintaining optimal brain function. (Atallah and Scanziani 2009; Yizhar et al. 2011). GABA (γ-aminobutyric acid) is the primary inhibitory neurotransmitter involved in both long-range and local inhibitory transmission. Changes in E/I balance are considered pathogenic factors for many neuropsychiatric disorders, including Alzheimer's disease and schizophrenia. (Snowden et al. 2019; Yang et al. 2016), primarily attributed to alterations in GABAergic signaling (Gonzalez-Burgos et al. 2015; Lozano et al. 2014). In mature neurons, inhibitory GABAergic signaling depends on intracellular chloride ion concentration. Under normal circumstances, intracellular Cl^−^ levels are low due to the outward transport of Cl^−^ by KCC2, leading to neuronal inhibition. However, our study found that overexpression of EZH2 significantly downregulates KCC2 expression, resulting in a transient elevation of intracellular Cl^−^ concentration and inhibition of GABAergic signaling, leading to a relatively excited state of neurons. If this state occurs in gliomas, it may trigger glioma-associated seizures. (Samudra et al. 2019). Cells may regulate this elevated intracellular Cl^−^ concentration state through other pathways to maintain homeostasis. We also note recent studies indicating that activation of the GABAergic system significantly enhances KCC2 activity (Heubl et al. 2017). Therefore, we hypothesize that EZH2 overexpression can suppress the expression of SLC12A5 and regulate the WNK1-OSR1-NKCC1 pathway through GABAergic activation. This may be a result of cells self-regulating to maintain internal homeostasis. We will focus on this aspect of research in future studies.

**Fig. 7:**
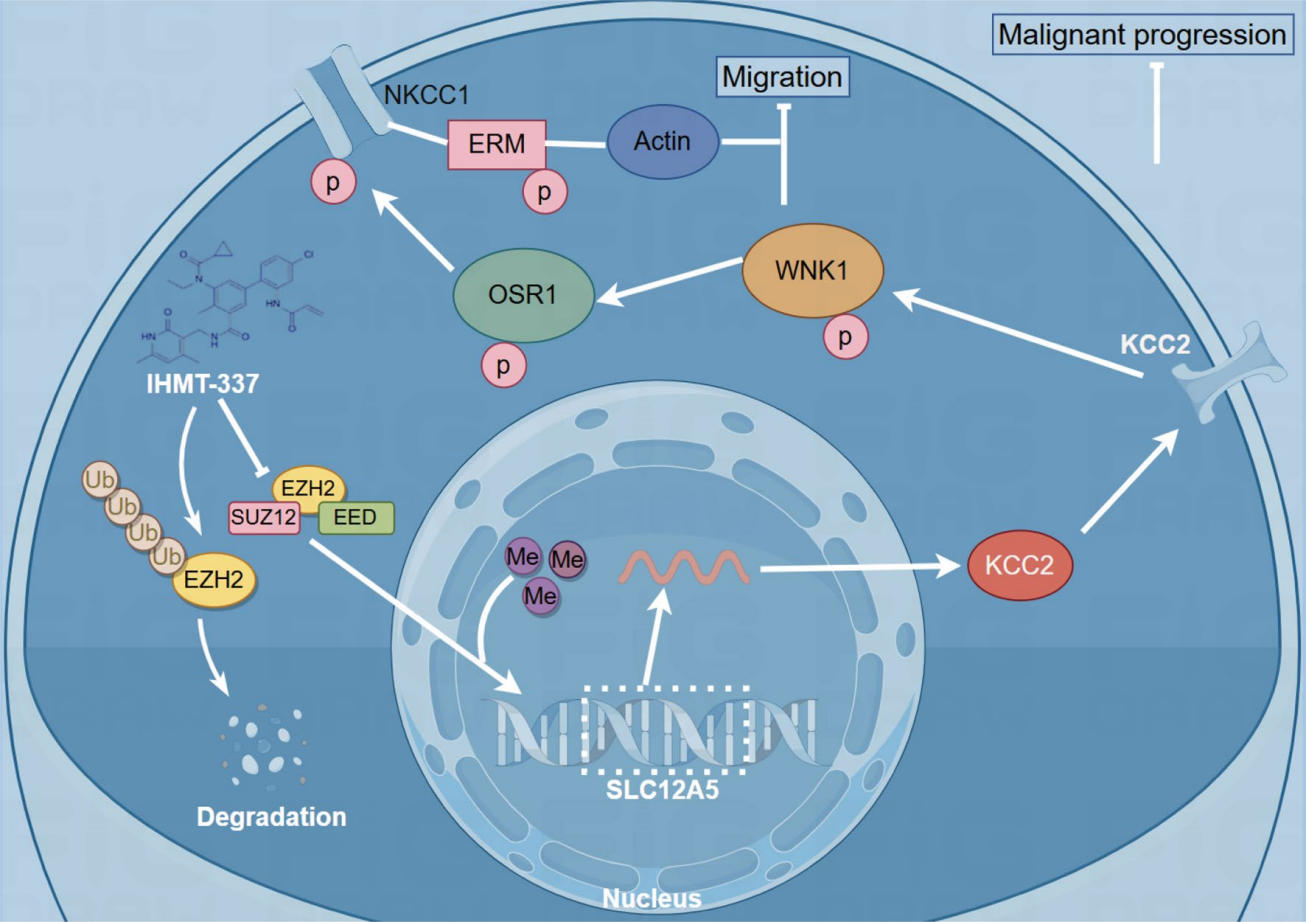
The possible mechanism of IHMT-337 targeting EZH2 to promote the expression of *SLC12A5* and inhibit glioma progression

In recent years, despite numerous failed clinical trials of new treatment regimens, recent research has shown promise in therapies such as targeted therapy and immune checkpoint inhibitors (Gai et al. 2022; Shi et al. 2022). IHMT-337, an EZH2-targeting inhibitor, has demonstrated efficacy in previous studies (Mei et al. 2023). However, its application in glioma treatment requires further investigation. Our study found that IHMT-337 efficiently degrades EZH2, inhibits WNK1 phosphorylation, and restores SLC12A5 expression at low concentrations. In vitro inhibits glioma migration, proliferation, and cell cycle progression. In vivo, IHMT-337 inhibits glioma growth in a mouse model, accompanied by EZH2 degradation and KCC2 expression restoration. These findings suggest a potential therapeutic mechanism for IHMT-337, depicted in Fig. 7, although the complex in vivo mechanism of EZH2 action warrants further investigation.

Our study again confirms the abnormal overexpression of EZH2 in gliomas, significantly correlated with patient prognosis. Additionally, EZH2 promotes glioma proliferation, migration, and invasion by suppressing the expression of *SLC12A5* through DNA promoter methylation. EZH2 can activate the WNK1-OSR1-NKCC1 cascade by regulating KCC2 expression, thereby regulating glioma migration. In addition, IHMT-337 targets EZH2 in vitro to inhibit WNK1 activation, thereby suppressing glioma migration. It also inhibits glioma cell proliferation and arrests cell cycle progression. IHMT-337 has the potential to penetrate the blood–brain barrier and inhibit glioma progression in vivo. This study expands our understanding of the mechanism of action of the EZH2-*SLC12A5* axis in gliomas, laying a new foundation for the clinical translation of IHMT-337 and providing new avenues for precise glioma treatment.

### Supplementary Information

Supplementary Material 1.Figure S1 (a) Analysis of EZH2's impact on prognosis in recurrent gliomas using the CGGA database. (b) Evaluation of EZH2's prognostic influence using the TCGA database. (c) Expression levels of *SLC12A5* in different types of gliomas. (d) Influence of *SLC12A5* on the prognosis of primary gliomas in the CGGA database. (e) Impact of *SLC12A5* on the prognosis of primary gliomas in the CGGA database. (f) Expression levels of *SLC12A5* in gliomas according to the TCGA database. (g) Influence of *SLC12A5* on patient prognosis according to the TCGA database.

Supplementary Material 2. Figure S2 (a) Methylation status of CpG islands in the *SLC12A5* gene, with the red box indicating the CpG island selected for experimental purposes. (b) Effects of KCC2 overexpression and knockdown on WNK1 activity in U251 cells. (c) Rescue of glioma cell migration promoted by EZH2 overexpression through KCC2 overexpression in U251 cells. (d) Degradation status of EZH2 by IHMT-337 in U251 cells.

Supplementary Material 3.Figure S3 (a) Flow cytometry analysis of the effect of IHMT-337 on apoptosis in U87 and U251 cells. (b) TUNEL assay to evaluate the effect of IHMT-337 on apoptosis in U87 and U251 cells. (c) EdU assay assessing the impact of IHMT-337 on cell proliferation in U251 cells. (d) CCK-8 assay to determine the effect of IHMT-337 on cell proliferation in U87 and U251 cells. (e) Flow cytometry analysis of the effect of IHMT-337 on the cell cycle in U251 cells.

Supplementary Material 4.

Supplementary Material 5.

Supplementary Material 6.

## Data Availability

The data supporting this study's findings are available from the corresponding author upon reasonable request.
