# Supplementary figures and images for: Unveiling the therapeutic potential of IHMT-337 in glioma treatment: targeting the EZH2-SLC12A5 axis
Source: Mol Med. 2024 Jun 17;30:91. doi: 10.1186/s10020-024-00857-0 (PMC11184773; doi:10.1186/s10020-024-00857-0)

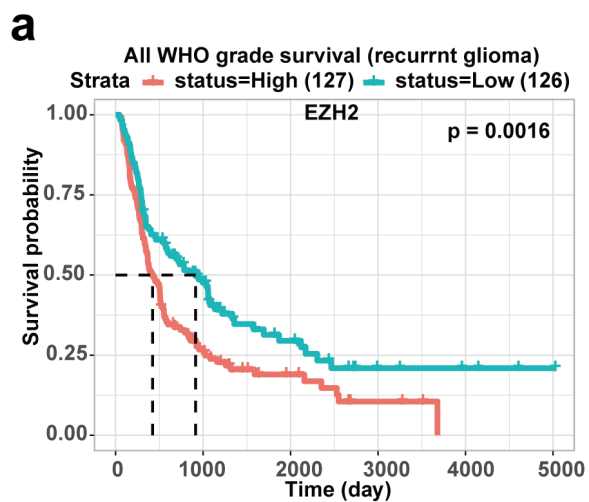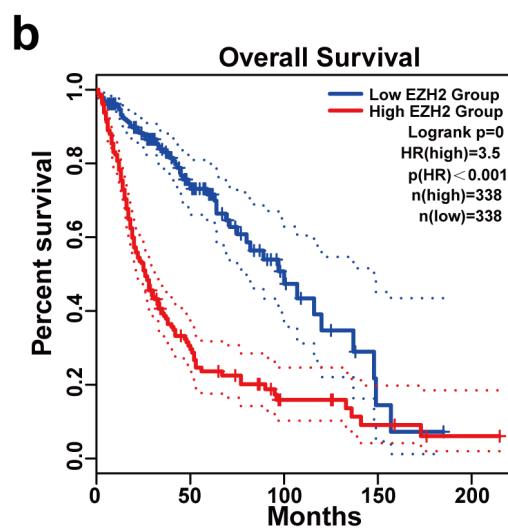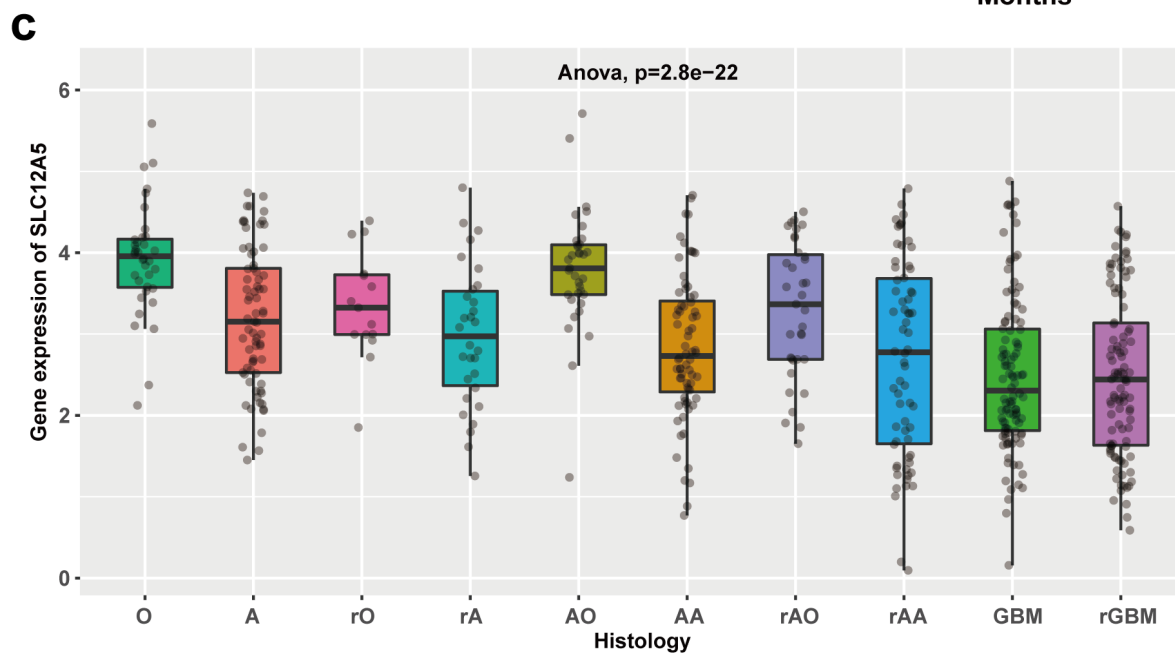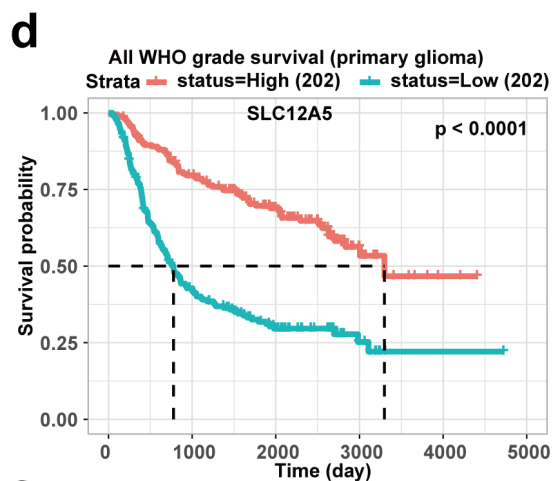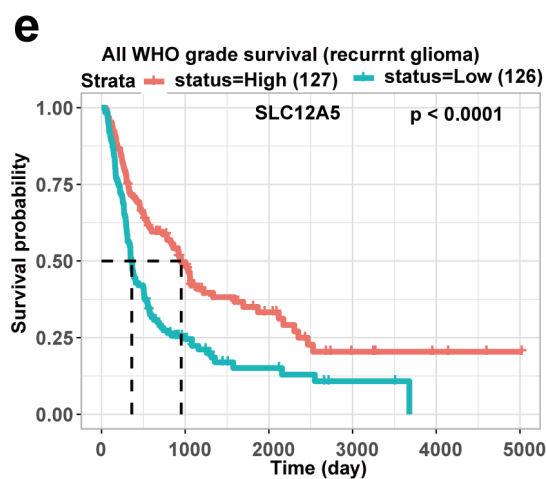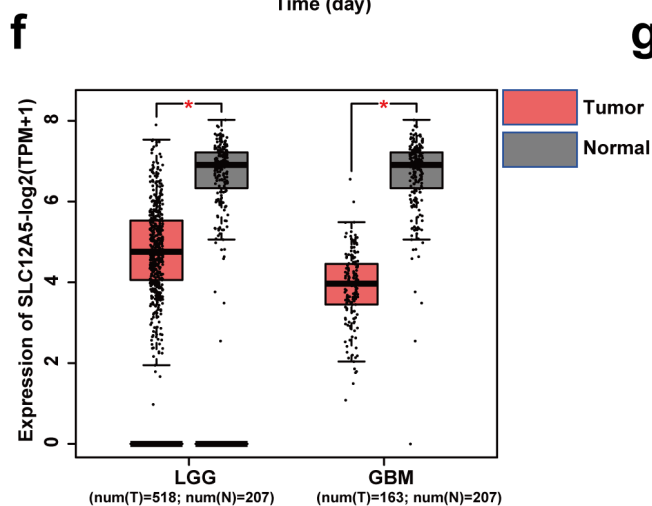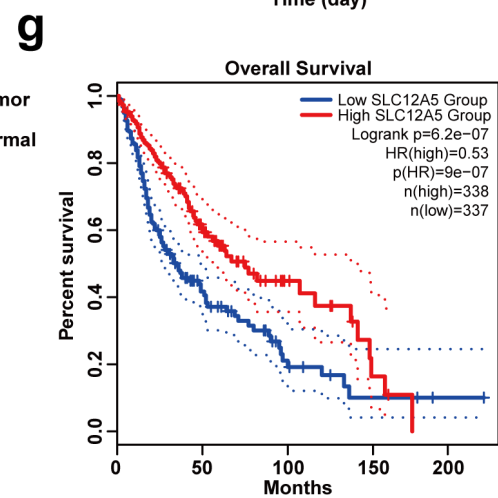

Supplement: Supplementary file 1 — Supplementary Material 1.Figure S1 (a) Analysis of EZH2's impact on prognosis in recurrent gliomas using the CGGA database. (b) Evaluation of EZH2's prognostic influence using the TCGA database. (c) Expression levels of SLC12A5 in different types of gliomas. (d) Influence of SLC12A5 on the prognosis of primary gliomas in the CGGA database. (e) Impact of SLC12A5 on the prognosis of primary gliomas in the CGGA database. (f) Expression levels of SLC12A5 in gliomas according to the TCGA database. (g) Influence of SLC12A5 on patient prognosis according to the TCGA database. [file 10020_2024_857_MOESM1_ESM.pdf]

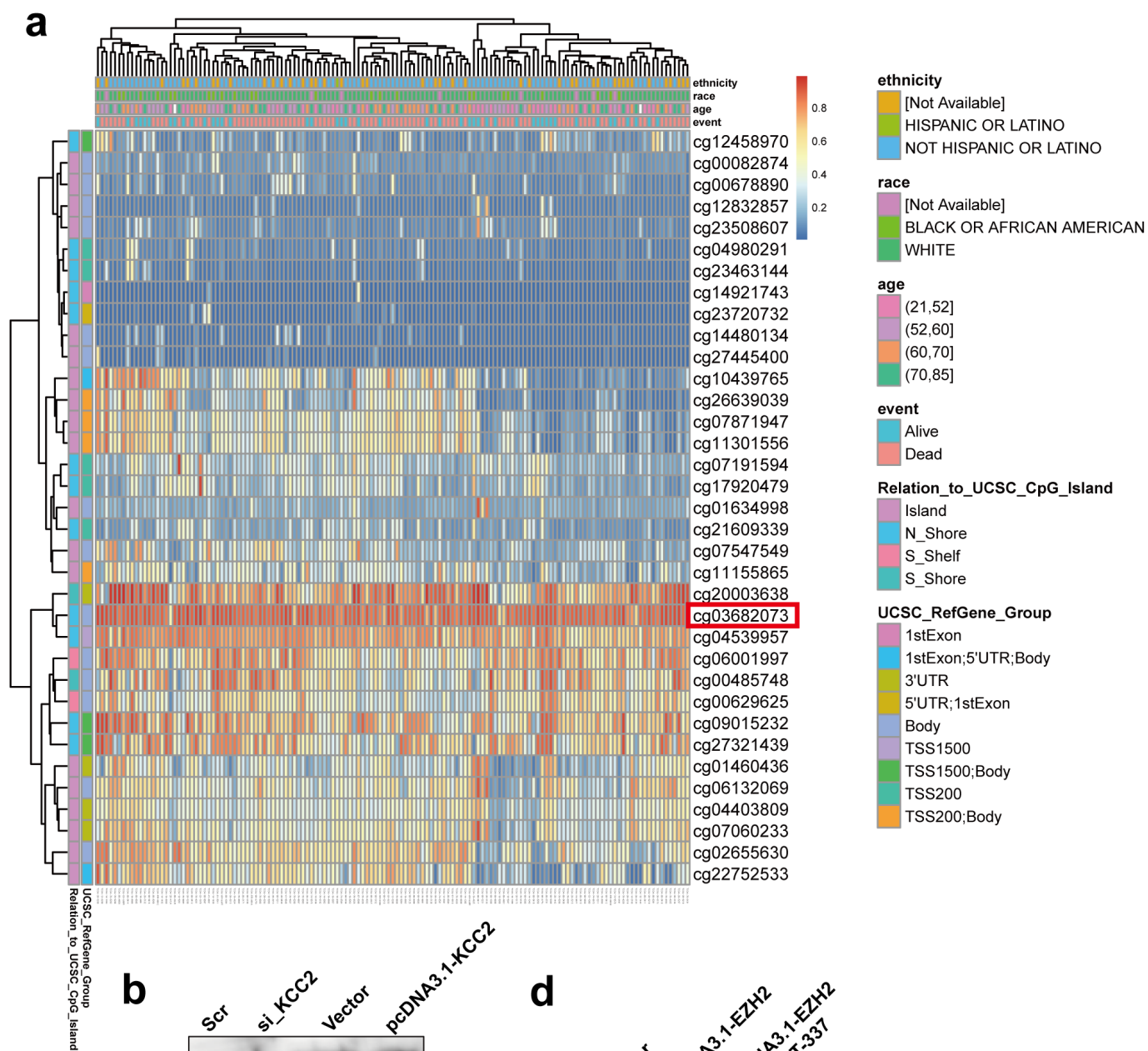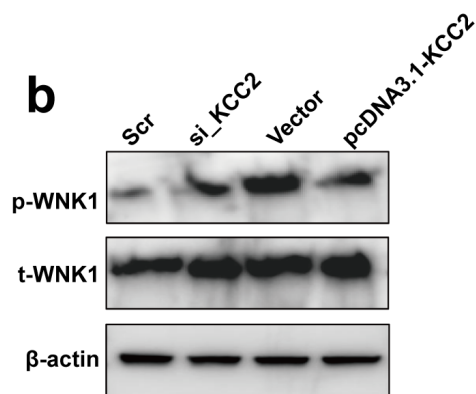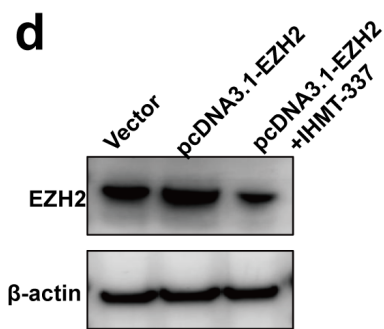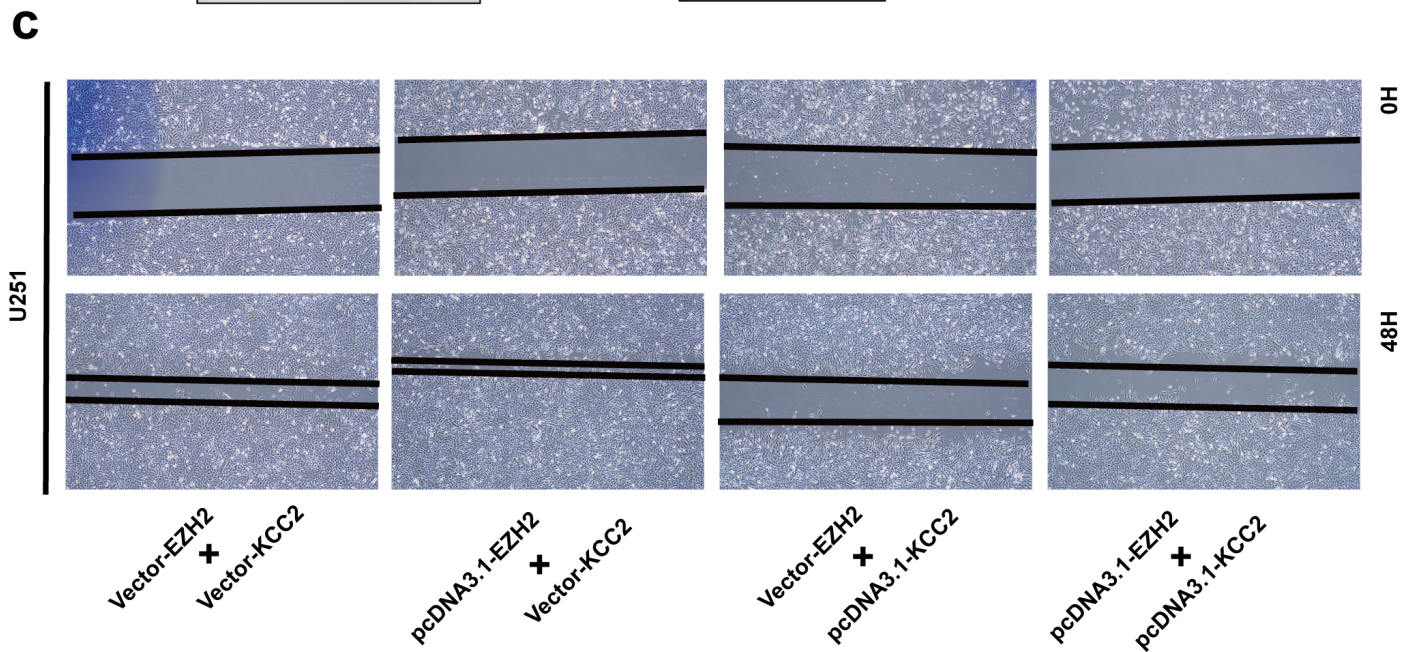

Supplement: Supplementary file 2 — Supplementary Material 2. Figure S2 (a) Methylation status of CpG islands in the SLC12A5 gene, with the red box indicating the CpG island selected for experimental purposes. (b) Effects of KCC2 overexpression and knockdown on WNK1 activity in U251 cells. (c) Rescue of glioma cell migration promoted by EZH2 overexpression through KCC2 overexpression in U251 cells. (d) Degradation status of EZH2 by IHMT-337 in U251 cells. [file 10020_2024_857_MOESM2_ESM.pdf]

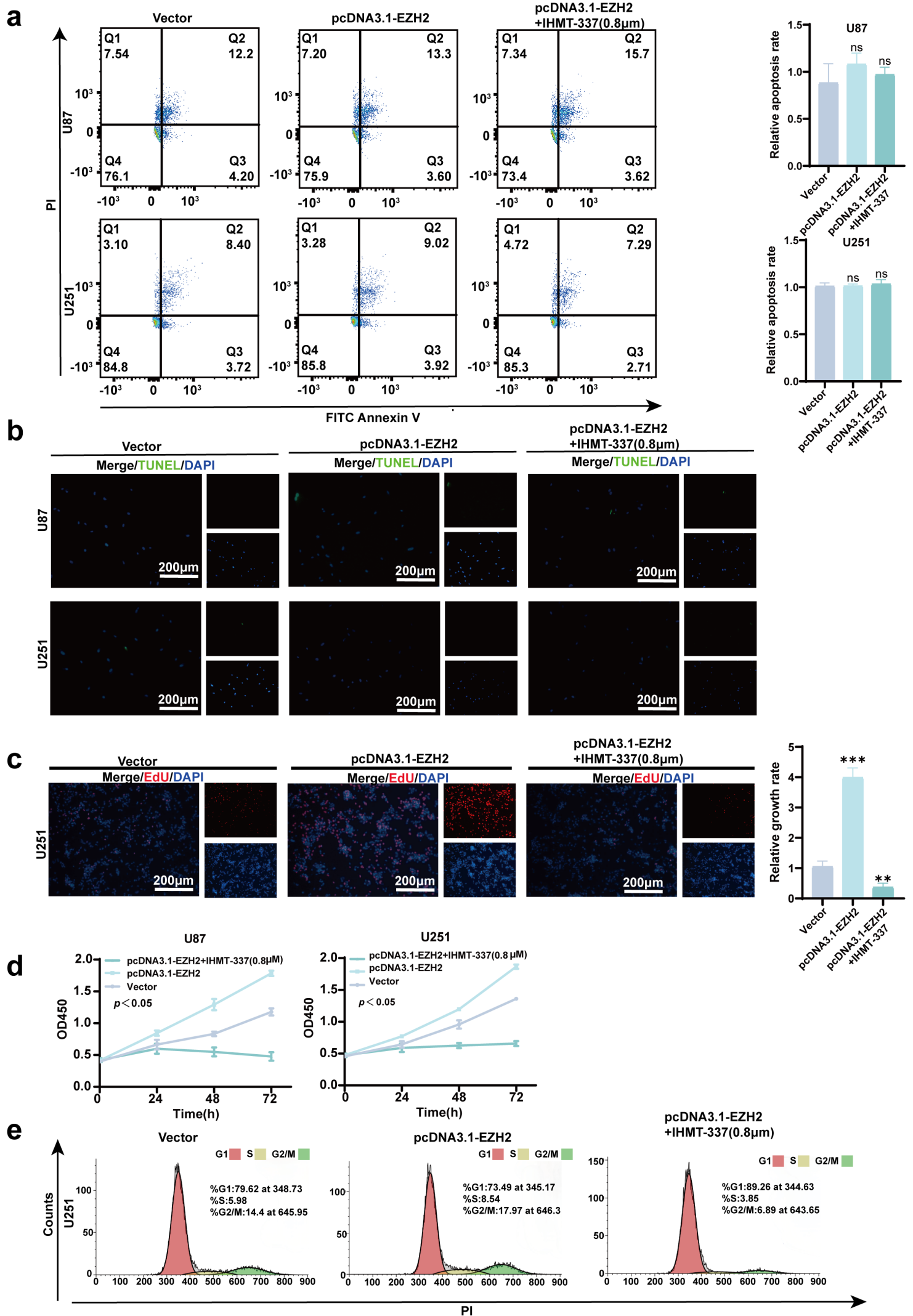

Supplement: Supplementary file 3 — Supplementary Material 3.Figure S3 (a) Flow cytometry analysis of the effect of IHMT-337 on apoptosis in U87 and U251 cells. (b) TUNEL assay to evaluate the effect of IHMT-337 on apoptosis in U87 and U251 cells. (c) EdU assay assessing the impact of IHMT-337 on cell proliferation in U251 cells. (d) CCK-8 assay to determine the effect of IHMT-337 on cell proliferation in U87 and U251 cells. (e) Flow cytometry analysis of the effect of IHMT-337 on the cell cycle in U251 cells. [file 10020_2024_857_MOESM3_ESM.pdf]
