# Supplementary material for: Unveiling the therapeutic potential of IHMT-337 in glioma treatment: targeting the EZH2-SLC12A5 axis
Source: Mol Med. 2024 Jun 17;30:91. doi: 10.1186/s10020-024-00857-0 (PMC11184773; doi:10.1186/s10020-024-00857-0)
Supplement: Supplementary file 4 — Supplementary Material 4. [file 10020_2024_857_MOESM4_ESM.docx]

| Name | Source | Item No. |
| --- | --- | --- |
| EZH2 Polyclonal antibody | Proteintech，China | 21800-1-AP |
| KCC2/SLC12A5 Polyclonal antibody | Proteintech，China | 28724-1-AP |
| ATP1A1 Polyclonal antibody | Proteintech，China | 14418-1-AP |
| NKCC1/SLC12A2 Polyclonal antibody | Proteintech，China | 13884-1-AP |
| Anti-phospho-NKCC1 antibody (Thr212/Thr217) | Sigma-Aldrich，Germany | ABS1004 |
| Anti-OXSR1 antibody | Abcam，UK | ab97694 |
| Anti-OXSR1 (phospho T185) antibody | Abcam，UK | ab192803 |
| WNK1 Polyclonal antibody | Proteintech，China | 28357-1-AP |
| Phospho-WNK1 (Thr60) antibody | Cell Signaling Technology,USA | 4946S |
| ERM Polyclonal antibody | Cell Signaling Technology,USA | 3142S |
| Phospho-ERM antibody | Cell Signaling Technology,USA | 3726S |
| Beta Actin Monoclonal antibody | Proteintech，China | 66009-1-Ig |
| HRP-conjugated Goat Anti-Rabbit IgG | Sangon biotech，China | D110058 |
| HRP-conjugated Goat Anti-Mouse IgG | Sangon biotech，China | D110087 |
| WNK1-IN-1 | MedChemExpress，USA | HY-151545 |
| Bumetanide | MedChemExpress，USA | HY-17468 |
| DAPI | Beyotime Biotechnology,China | P0131 |
| One Step TUNEL Apoptosis Assay Kit | Beyotime Biotechnology,China | C1086 |
| BeyoClick™ EdU Cell Proliferation Kit with Alexa Fluor 647 | Beyotime Biotechnology,China | C0081S |
| DAB (SA-HRP) Tunel Cell Apoptosis Detection Ki | Servicebio Technology CO.,LTD，China | G1507 |
| BD Pharmingen™ FITC Annexin V Apoptosis Detection Kit I | BD biosciences,USA | 556547 |

Supplementary Table 1 Materials used in the study
