# Supplementary material for: Unveiling the therapeutic potential of IHMT-337 in glioma treatment: targeting the EZH2-SLC12A5 axis
Source: Mol Med. 2024 Jun 17;30:91. doi: 10.1186/s10020-024-00857-0 (PMC11184773; doi:10.1186/s10020-024-00857-0)
Supplement: Supplementary file 5 — Supplementary Material 5. [file 10020_2024_857_MOESM5_ESM.docx]

Supplementary Table 2-1: All RT-qPCR primers used in this study

| Primer name | Sequence ( 5'-3' ) | Source |
| --- | --- | --- |
| WIF1 | CCGAAATGGAGGCTTTTGTA TGGTTGAGCAGTTTGCTTTG | The primers were designed by Primer3 ( https://bioinfo.ut.ee/primer3-0.4.0/) and synthesized by Shanghai Sangon Biotechnology Co., Ltd. |
| SVOP | CTGCCGACATCAAGAACTCA GGTGACTCTGGGTGGTGTCT |  |
| PACSIN1 | CATCCATGTGTACCGTGAGC  CTCCTTCTTGGTGGTGGTGT |  |
| RBFOX1 | TGCTTTTGCACCTTTGACTG AGCTTCCTTTCTCCCCACAT |  |
| CCK | GGGTGACTGACTAGGGGTGA CTCGCAGTTCTCCAGGTTTC |  |
| NEFL | GAAGAGGAGGCAGCTGAAGA  AAGGAAATGGGGGTTCAATC |  |
| PHYHIP | AGAGCTGTCCCTCCTTCTCC  AGACCCCTGCACTCTAAGCA |  |
| MOBP | GCTCCTGGACTCATTGCTTC CAGAGATAGCCCTGCTCACC |  |
| GABRG2 | CTGGTGGAGTATGGCACCTT TGCCGTCCAGACACTCATAG |  |
| SLC12A5 | CAATCCGGAGAAAGAATCCA ACTTGTCCTTGGTCCAGGTG |  |
| KCNS1 | GGAGGGAAGGAGGATGAGTC GCAGGGATTGGACCACTTTA |  |
| CABP1 | CCTTTGGCCAGGATAGATCA GGAGTTTAGGCCCCATTAGC |  |
| RBFOX3 | GCATCCAACCAAGTCCAAGT TGGGAAAGGAAGACTGGATG |  |
| SYNPR | GGCATTGATTGGTGACTCCT AAGCTGATGAACCCACCAAC |  |
| BIRC5 | GCCTTTCCTTAAAGGCCATC AACCCTTCCCAGACTCCACT |  |
| CENPA | TCCGAAAGCTTCAGAAGAGC GCACATCCTTTGGGAAGAGA |  |
| HIST1H1B | AAATCCCCGGCTAAGAAGAA TCTTAAGGGCTGCCAAAGAA |  |
| KIF18B | CTCCAGGGCTACAACTGCTC CCAGGAGGTCATGGATCTGT |  |
| CDCA2 | TTTGAAGCACCTGCCTTTCT CAGGAACAGGTGACTGCTCA |  |
| ESM1 | GCCCTTCCTTGGTAGGTAGC TGTTTCCTATGCCCCAGAAC |  |
| NDC80 | TCCTCATACATGGCCTCACA TGTCGGCACCACTCATAAAA |  |
| NCAPG | TTCAAGGCTGGTTACGGTTC AGGGCACACCAATACAAAGC |  |
| KIF14 | CCAATGCTATCAGCAGCAAA TGTAATGTCGGGTTCCCATT |  |
| COL3A1 | TACGGCAATCCTGAACTTCC GTGTGTTTCGTGCAACCATC |  |
| HOXA7 | GGCACCCCCAAACTACCTAT GGCACTAGGTAGCAGGCAAG |  |
| MMP19 | AAACCTGGATGCTGCTGTCT TTGAGGCGCCAGTAGACTTT |  |
| MELK | ATGATCACCTCACGGCTACC TGCAGGTGTTCTGCATAAGG |  |
| CENPF | GTCAGCGACAAAATGCAGAA TGCATATTCTTGGCTTGCTG |  |
| GAPDH | CGACCACTTTGTCAAGCTCA AGGGGTCTACATGGCAACTG |  |
| EZH2 | AGGACGGCTCCTCTAACCAT CTTGGTGTTGCACTGTGCTT |  |

Supplementary Table 2-2 : All si-RNA sequences used in this study

| siRNA name | Sequence ( 5'-3' ) | Source |
| --- | --- | --- |
| si-SLC12A5 | CAUCAGACGUGGAGGAACUTT AGUUCCUCCACGUCUGAUGTT | siRNA and its control products  were designed and synthesized by Nanjing Corues Biotechnology Co., Ltd. |
| si-NC（SLC12A5） | UUCUCCGAACGUGUCACGUTT ACGUGACACGUUCGGAGAATT |  |
| si-EZH2 | GCUCAAGAGGUUCAGACGATT UCGUCUGAACCUCUUGAGCTT |  |
| si-NC（EZH2） | UUCUCCGAACGUGUCACGUTT ACGUGACACGUUCGGAGAATT |  |

Supplementary Table 2-3 : All MS-PCR sequences used in this study

| Primer name | Sequence ( 5'-3' ) | Source |
| --- | --- | --- |
| SLC12A5(M) | GGGTTTGGTGATGGAAAGGTC AACCAACCTAATCCCGCCG | The primers were designed and synthesized by **Wuhan Servicebio Technology CO.,Ltd** |
|  |  |  |
| SLC12A5(U) | GGGTTTGGTGATGGAAAGGTT CAAACCAACCTAATCCCACCA |  |
|  |  |  |
