# Supplementary material for: Unveiling the therapeutic potential of IHMT-337 in glioma treatment: targeting the EZH2-SLC12A5 axis
Source: Mol Med. 2024 Jun 17;30:91. doi: 10.1186/s10020-024-00857-0 (PMC11184773; doi:10.1186/s10020-024-00857-0)
Supplement: Supplementary file 6 — Supplementary Material 6. [file 10020_2024_857_MOESM6_ESM.docx]

| Supplementary Table 3:The number of samples examined via IHC for EZH2 | | |
| --- | --- | --- |
| Group | Category | samples |
| Normal( non-tumor tissue ) | Encephalorrhagia | 8 |
|  | Epilepsy | 2 |
| LGG（Low-grade glioma） | WHOⅠ | 5 |
|  | WHOⅡ | 8 |
| HGG（High-grade glioma） | WHOⅢ | 15 |
|  | WHOⅣ | 20 |
